# Glycopolymer Grafted Silica Gel as Chromatographic Packing Materials

**DOI:** 10.3390/ijms20010010

**Published:** 2018-12-20

**Authors:** Gaoqi Ma, Xitao Luo, Xitong Sun, Weiyan Wang, Qinghui Shou, Xiangfeng Liang, Huizhou Liu

**Affiliations:** 1 School of Chemical Engineering, Xiangtan University, Xiangtan 411105, China; Magq@smail.xtu.edu.cn; 2 CAS Key Laboratory of Bio-Based Materials, Qingdao Institute of Bioenergy and Bioprocess Technology (QIBEBT), Chinese Academy of Sciences (CAS), Qingdao 266101, China; luoxt@qibebt.ac.cn (X.L.); sunxt@dicp.ac.cn (X.S.); hzliu@ipe.ac.cn (H.L.); 3 University of Chinese Academy of Sciences, Shijingshan District, Beijing 100049, China

**Keywords:** glycopolymers, ATRP, SiO_2_-g-GAEMA, packing materials

## Abstract

The modification of the surface of silica gel to prepare hydrophilic chromatographic fillers has recently become a research interest. Most researchers have grafted natural sugar-containing polymers onto chromatographic surfaces. The disadvantage of this approach is that the packing structure is singular and the application scope is limited. In this paper, we explore the innovative technique of grafting a sugar-containing polymer, 2-gluconamidoethyl methacrylamide (GAEMA), onto the surface of silica gel by atom transfer radical polymerization (ATRP). The SiO_2_-g-GAEMA with ATRP reaction time was characterized by Fourier infrared analysis, Thermogravimetric analysis (TGA), and elemental analysis. As the reaction time lengthened, the amount of GAEMA grafted on the surface of the silica gel gradually increased. The GAEMA is rich in amide bonds and hydroxyl groups and is a typical hydrophilic chromatography filler. Finally, SiO_2_-g-GAEMA (reaction time = 24 h) was chosen as the stationary phase of the chromatographic packing and evaluated with four polar compounds (uracil, cytosine, guanosine, and cytidine). Compared with unmodified silica gel, modified silica gel produces sharper peaks and better separation efficiency. This novel packing material may have a potential for application with highly isomerized sugar mixtures.

## 1. Introduction

The synthesis of sugar-based monomers and polymers has been widely reported over the last two decades. The carbohydrate residues confer high hydrophilicity and water solubility and they are widely applied in macromolecular drugs [1], drug delivery [2], cell targeting, and adhesion [3,4]. Use of such polymers as stationary in separation applications and bioassays has also been suggested for structure-controllable sugar polymers [5]. Glycopolymers, defined as functional polymer materials comprising sugar moieties as pendant or terminal groups, have attracted great attention as model systems to study the specific molecular recognition functions of saccharides [6]. The side glycosyl groups are composed by one, two or more saccharides including monosaccharide, disaccharide, or oligosaccharide, or they comprise a synthetic polymer. Compared with natural sugar complexes, glycopolymers have a well-defined structure and composition, and they can be chemically designed and synthesized based on the actual scientific issues, such as hydrogels [7], degradable materials [8], biomedical materials [9].

A wide range of applications of glycopolymers with the feature of pendent carbohydrate moieties exist, so it is necessary to control the structure with respect to attributes such as glycosylation density, position, and molecular weight. Controlling the molecular chain length of sugar-containing polymers is also necessary to achieve the polymer’s desired properties in a particular application [10]. Except for the large distribution of the hydroxyl group, the fraction of amide is the only highly distributed moiety in the polymer grafted onto the surface of silica particles. Both hydroxyl and amide bonds are functional groups with a strong polarity. The sugar-containing polymers possessing the above characteristics have potential applications for hydrophilic interaction liquid chromatography (HILC). HILC, one chromatographic mode with good separation ability of strongly polar compounds, was first proposed in 1990 [11]. The binding of monosaccharides [12], maltose, isosorbide [13], and oligosaccharides [14] on the surface of silica gel has also been reported. However, these natural sugar structures are only suitable for analysis in specific fields. It is also meaningful to develop a synthetic sugar-containing polymer. In chromatography, extreme differences in polarity could be produced, which is critical to the separation of agents with a similar structure. In this work, glycopolymers were grafted onto the surface of the silica gel for further use as packing materials.

**Figure 1:**
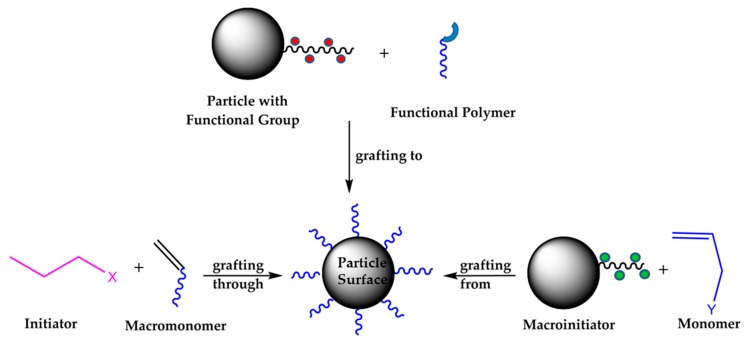
Three main strategies for grafting polymers on solid surfaces: Grafting to, grafting through, and grafting from. The terminal double bond, one typical polymerization unit, is drawn.

Generally, three methods are available to covalently graft polymer chains onto a silica surface: (1) The “grafting to” technique, where polymers are first synthesized and then introduced onto the surface through binding between the polymers’ reactive end group and the surface functional group of the silica; (2) the “grafting through” method, where a macromonomer with a functional terminal group is synthesized, and the end groups are polymerized to form the main chain; (3) and the “grafting from” approach, where the polymer chain grows from an initiator-functionalized self-assembled monolayer or initiator-modified particle surface [15]. A mechanism diagram of the three grafting modes is shown in Figure 1. Preparation of polymers with a high grafting density by using grafting to techniques is quite difficult because of the steric hindrance imposed by dense grafting. Although by using grafting through methods one can obtain a covalently bound side chain on each repeating unit of the backbone, the concentration of the polymerization end group is necessarily low, and the steric hindrance at the end of the propagation chain is large, resulting in a slow polymerization process and relatively low conversion rates [16]. Instead, using grafting from approaches can involve growth of polymer chains from solid surfaces by means of surface-initiated polymerization (SIP) of monomers. However, limitations arise from a synthetic perspective of this method (e.g., high polymer dispersity index—PDI) [17].

**Scheme 1:**
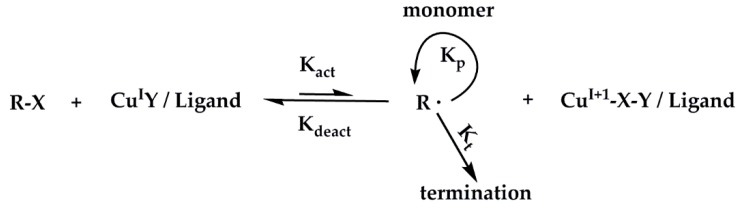
Reaction mechanism of atom transfer radical polymerization (ATRP).

In the methods described above, shortage in polymer structure, dispersion, and terminal functional groups can be compensated for by the atom transfer radical polymerization (ATRP) methods pioneered by Matyjaszewski [18]. Among the controlled polymerization techniques, ATRP has attracted a great deal of attention in the past few years. Generally, the reaction proceeds with organic halogens as the initiator and transition metal salts such as CuBr/PMDETA as the catalyst (see Scheme 1). During the polymerization process, the halogen atom is reversibly transferred and related to the dormant polymer chain. The addition of the transition metal and the ligand (L) catalyst can cause the dormant species to lose the halogen atom and generate an alkoxy radical active chain and oxidation state catalyst, which form a propagating radical and deactivator complex by a reversible halogen–atom transfer reaction. During the reaction, by controlling the concentration of the free radical active species, the side reaction caused by the irreversible bimolecular termination can be reduced, to better control the polymerization reaction.

Taking these factors into consideration, we constructed a glycopolymer (2-gluconamidoethyl methacrylamide—GAEMA) [19] grafted onto a silica surface. This was used as a packing material for high performance liquid chromatography (HPLC) in separating model materials, including uracil, adenosine, cytosine, and cytidine. GAEMA contains a high density of amide and hydroxyl groups, making it attractive as a polar stationary phase because of its high hydrophilicity of these groups. Because of the dual binding between the amide and hydroxyl groups with the polar solutes in the mobile phase, the two structurally similar isomers may be effectively separated.

## 2. Results and Discussion

### 2.1. SiO_2_-g-poly (2-gluconamidoethyl methacrylamide)

**Scheme 2:**
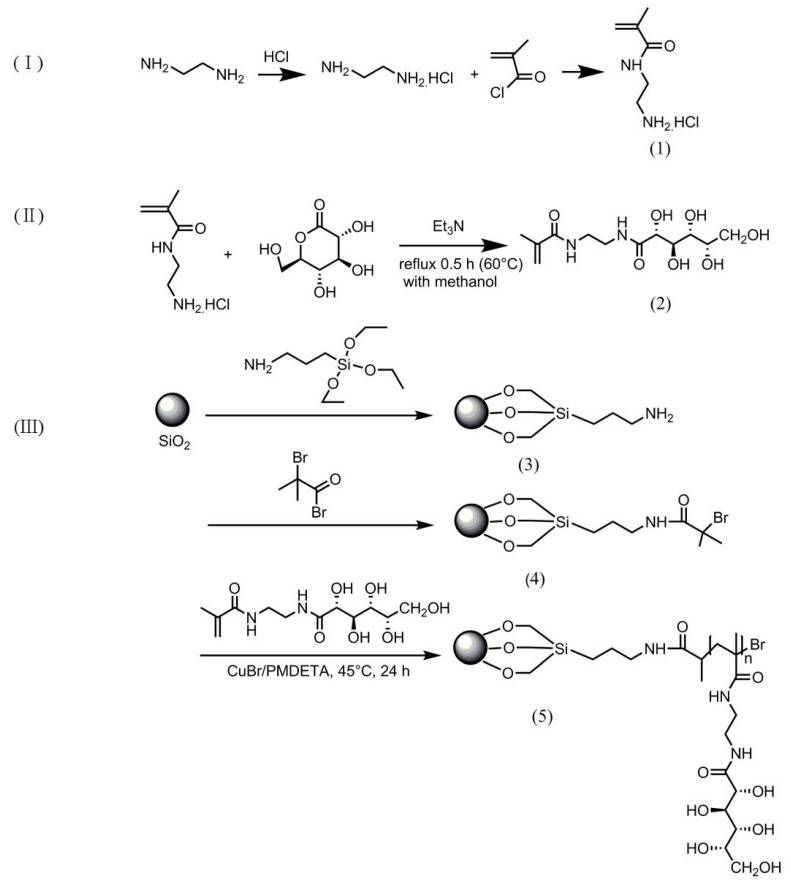
Synthesis steps for the preparation of SiO_2_-g-GAEMA.

The GAEMA monomer was synthesized without using any protecting group by Narain et al. [5,20]. For the first time, we reported the grafting of the sugar-containing monomer GAEMA onto the surface of silica gel by ATRP. During the polymerization, Cu^2+^ was introduced by adding a small amount of copper chloride to act as a deactivator, reducing the reaction rate and making the reaction controllable [21]. The glycomonomers were characterized by ^1^H and ^13^C nuclear magnetic resonance (NMR) spectra. The structural characterization of Scheme 2 (5) was carried out by spectroscopic (Fourier Transform infrared spectroscopy—FTIR), and thermoanalytical methods (thermogravimetric analysis—TGA). The surface grafting ratio of the silica gel was calculated by elemental analysis.

### 2.2. IR Analysis

**Figure 2:**
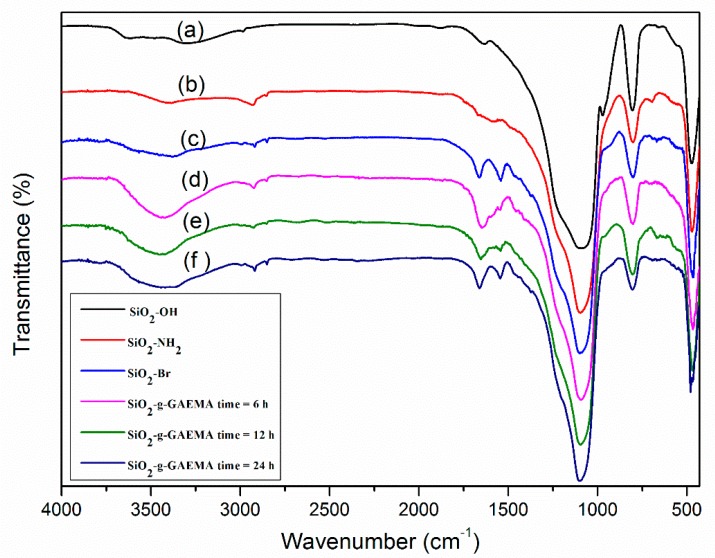
The FT-IR spectra of (a) bare SiO_2_, (b) SiO_2_-NH_2_, (c) SiO_2_-Br, (d) SiO_2_-g-GAEMA (reaction time = 6 h), (e) SiO_2_-g-GAEMA (reaction time = 12 h), and (f) SiO_2_-g-GAEMA (reaction time = 24 h).

Figure 2 shows the FT-IR spectra of (a) bare SiO_2_, (b) SiO_2_-NH_2_, (c) SiO_2_-Br, (d) SiO_2_-g-GAEMA (reaction time = 6 h), (e) SiO_2_-g-GAEMA (reaction time = 12 h), and (f) SiO_2_-g-GAEMA (reaction time = 24 h). For bare SiO_2_ particles, 1089.08 cm^−1^ belonged to the stretching vibration peak of Si-O-Si; 3302.00 cm^−1^ was attributed to the free hydroxyl vibration peak (which is associated with O-H stretching vibrations of the hydroxyl group on the silica surface); and the peak at 971.88 cm^−1^ came from Si-OH vibrations [22]. In Figure 2b, the missing Si-OH peak at 971.88 cm^−1^ was replaced by an amino group. According to TGA, the residual weight of the bare silicon sphere was 94.0% at 600 °C, whereas the SiO_2_-Br was 89.8%, indicating that 10.2% of 2-bromoisobutyryl bromide was successfully grafted. As shown in Figure 2c, peaks at around 1661.2 cm^−1^ and 1541.3 cm^−1^ of SiO_2_-Br are ascribed to the stretching vibration of C=O and N-H in NH-O=C groups, corresponding to amide I and II bands, respectively. In the spectra of SiO_2_-Br and SiO_2_-g-GAEMA, new peaks at 2925.2 cm^−1^ and 2879.0 cm^−1^ of C-H in CH_2_ and CH_3_ were observed because the organic chain was introduced [23]. After the reaction, the remaining SiO_2_-g-GAEMA weights were 87.2% (reaction time = 6 h), 86.6% (reaction time = 12 h), and 75.7% (reaction time = 24 h) at 700 °C, which shows that the grafting percentage of GAEMA was 12.8%, 13.4% and 24.3%, respectively. The vibration peak of C-H increased with increasing reaction time, while the stretching vibration peak of the amide bond became obvious (see Figure S3), which may be related to the increase in grafting density of the sugar-containing polymer on the surface of the silica gel.

### 2.3. TGA Analysis

**Figure 3:**
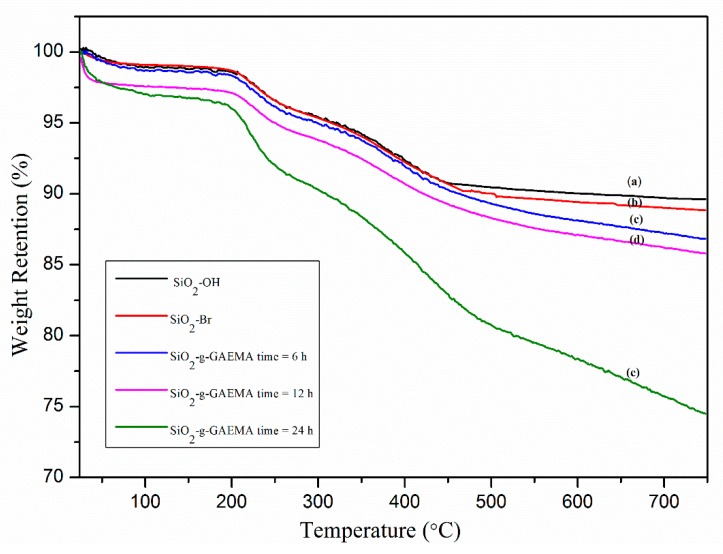
Thermogravimetric analysis (TGA) curves of (a) bare SiO_2_, (b) SiO_2_-Br, (c) SiO_2_-g-GAEMA (reaction time = 6 h), (d) SiO_2_-g-GAEMA (reaction time = 12 h), and (e) SiO_2_-g-GAEMA (reaction time = 24 h).

Results from TGA analysis of the synthesized nanocomposites are shown in Figure 3. All samples showed significant weight loss at 50–180 °C, due to evaporation of the bound water on the surface. However, the bare SiO_2_ spheres lost more weight than the modified SiO_2_, possibly accounted for by surface agglomeration of the bare SiO_2_ sphere particles and more hydrogen bonding, resulting in the adsorption of more water molecules [23]. Another weight loss phase of the bare silicon balls occurred at 450–560 °C, which may be due to the dehydroxylation of the Si-OH surface. Figure 3b shows the weight loss curve of SiO_2_-Br. Although the initiator SiO_2_-Br has a wide range of weight loss, it is particularly pronounced at 210–280 °C [24] because of the polarity and thermodynamic instability of the C-Br bond on its surface (it may release Br_2_ or HBr) [25]. For SiO_2_-g-GAEMA, the C-Br bond decomposed with the onset at 200 °C, and the GAEMA decomposed with the onset at 340 °C. The polymers grafted onto the silica surface can be approximately estimated to be 9% (reaction time = 6 h), 11% (reaction time = 12 h), and 21% (reaction time = 24 h), respectively. Weight loss increased with reaction time, verifying that the GAEMA was grafted onto the silica surface in a controllable manner.

### 2.4. Characterization of GAEMA Grafted Silica Particles

Table 1 shows that the ratio of C, N, and H in the brominated silica gel significantly increased compared with the bare silicon sphere, further demonstrating the synthesis of the macro-initiator SiO_2_-Br. Table 2 shows that the grafting amount gradually increased with the extension of the reaction time, and the grafting amount was largest at 24 h. The grafting amount did not increase further, even when the reaction time was prolonged past 24 h. It is possible that this occurred because the concentration of the radical was kept constant during the polymerization. The radical-induced bimolecular termination and the irreversible chain-transfer reaction were limited, showing the characteristics of living polymerization [26].

By linking FT-IR analysis, TGA analysis, and elemental analysis, we validated a process in which the surface of the silica gel was grafted with the sugar-containing polymer GAEMA.

### 2.5. Chromatographic Performance of SiO_2_-g-GAEMA

**Figure 4:**
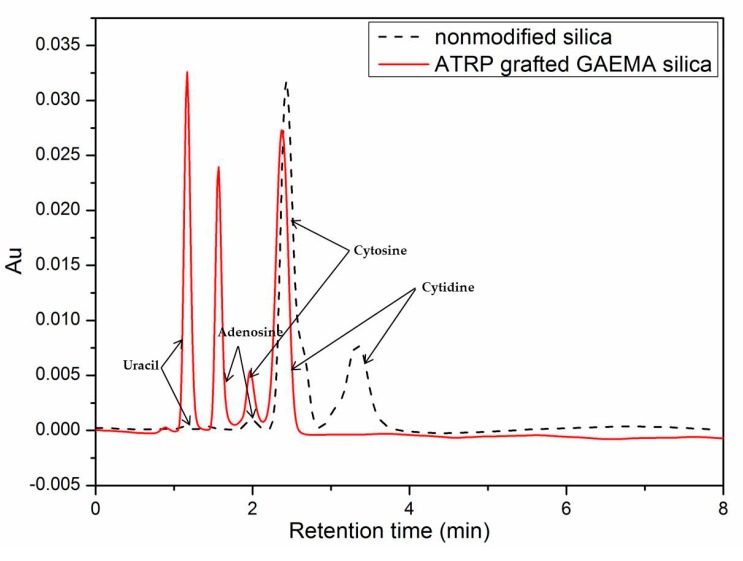
Hydrophilic interaction liquid chromatography (HILC) mode separation of uracil (200 ppm), adenosine (100 ppm), cytosine (50 ppm) and cytidine (200 ppm). Mobile phase: Acetonitrile/H_2_O (90:10); flow rate: 1 mL/min; column temperature: 30 °C; UV detection (254 nm). Dashed line is unmodified silica. Solid line indicates GAEMA grafted silica.

Finally, we selected a SiO_2_-g-GAEMA with a grafting amount of 1.36 mg/m^2^ as the chromatographic packing material, and packed the column according to the method described in Section 3.8. The chromatographic evaluation was conducted with four polar compounds (uracil, adenosine, cytosine, and cytidine). These hydrophobic probes are widely used for chromatographic evaluation [27]. Compared with bare silicon spheres, the silica gel grafted with GAEMA had a better peak shape and good efficiency in separating all compounds (as depicted in Figure 4). The chromatographic peak is considered highly symmetrical if the symmetry factor is in the range of 0.8–1.2 [28]. As shown in Table 3, the symmetry factor of the 200 ppm uracil in the GAEMA grafted silica column (0.98) was larger than that of the unmodified silica column (0.75). The calculated N values were 3960 and 8258 plates/m for the unmodified and modified silica column, respectively. To obtain sharp, narrow peaks, and good resolution, a higher N value is commonly needed. This indicates that room for improvement persists in the hydrophilic interaction with the chromatographic stationary phase.

## 3. Materials and Methods

High quality porous silica (5 µm, 100 Å) was used as the grafting substrate for all experiments. Both 3-Aminopropyl triethoxysilane (APTES) and N, N, N’, N’’, N’’-pentamethyl diethyenetriamine (PMDETA) were obtained from Aladdin (Shanghai, China). Toluene (SINOPHARM, 99+%, China) was distilled from sodium/benzophenone and stored under argon when not used. Methacryloyl chloride (Sigma-Aldrich, St. Louis, MO, USA) was filtered through basic alumina and distilled prior to use. Trichloromethane (Sinopharm Chemical Reagent Co., 99+%, Shanghai, China) is treated with anhydrous sodium sulfate and then distilled under reduced pressure. HPLC-grade methanol and acetone were obtained from Merck KGaA. Copper (I) bromide was purchased from SIGMA-ALDRICH and purified via stirring in acetic acid, washed with ethanol, and then dried in vacuo. Triethylamine (TEA, Alfa Aesar, 99+%, Shanghai, China) was distilled over potassium hydroxide. The 2-bromoisobutyryl bromide (Aladdin) was used as received. Ethylenediamine (MACKIN, 99+%, China) was dried over calcium oxide and potassium hydroxide, and distilled before use following the method described by Perrin and Armarego [29]. Water was prepared in Ultra equipment (PURELAB Classic UV, Paris, France) and had a resistivity of 18.2 MΩcm^−1^. Cytosine was purchased from Aladdin, cytidine was purchased from Sigma (St. Louis, MO, USA), uracil was from Macklin (Shanghai, China), and adenosine was Aladdin (Shanghai, China).

### 3.1. Instrumentation

IR measurements were conducted on a Thermo Fisher Nicolet (Grand Island, NY, USA) FTIR-6700 spectrometer in KBr pellets at room temperature. TGA was performed on an STA–449F5 thermogravimetric analysis (Netzsch, Bavaria, Germany) using a temperature 20–750 °C of 10 °C/min in a nitrogen atmosphere flow. Elemental analyses were carried out on an GmbH Vario EL cube apparatus (Hanau, Germany). ^1^HNMR spectra were measured with a Bruker AVANCE III 600 MHZ spectrometer with D_2_O as the solvent. Chromatographic performance evaluations experiments were carried out with a Waters 1525 HPLC (Milford, MA, USA) and a Waters 2707 autosampler, a 2707 column compartment, a Waters 2489 UV/visible detector, and a Waters 1525 binary gradient pump.

### 3.2. Synthesisof 2-Aminoethyl Methacrylamide Hydrochloride (AEMA, Scheme 2 (1))

AEMA was synthesized according to a previously reported method that was modified [30]. First, 10.1 mL of 15 mmol ethylenediamine was dissolved in 150 mL ultrapure water at 0 °C; the pH was adjusted to 8.5 by adding 74 mL of 3N HCl solution. Methacryloyl chloride (16.5 mmol, 16.1 mL) diluted with anhydrous chloroform (100 mL), was then added to the aqueous ethylenediamine solution dropwise over 2 h. After the addition, the reaction continued for another 2 h at 0 °C. The aqueous and organic layers were separated, and the aqueous layer was extracted with anhydrous chloroform 3 times (3 × 50 mL) to remove the unreacted methacryloyl chloride [31]. Following this, the aqueous layer was purified and concentrated under reduced pressure to obtain a white solid. The residue was washed with room temperature methanol repeatedly and filtered through a funnel. The purification is very important to successfully obtain a high purity product. Using the method of Narain et al. [32], the reaction proceeded at −30 °C, but the temperature was not easy to control and the product could not be easily purified in the recrystallization and concentration stages. On the other hand, following the method of Solomon et al. [30], the reaction proceeded at about 0 °C, with a slow reaction rate and a slow yield. Herein, the reaction was held at −5 °C and purified in liquid methanol. At the end of the concentration process, traditional filtration could be used instead of rotary steaming, which greatly reduced energy consumption and effectively increased the yield. The filtrate was finally concentrated to obtain a clear yellow oil. Finally, the solvent alone was removed under vacuum. The final product was a light-yellow powder (58% yield). The product was characterized by ^1^H and ^13^C NMR. AEMA: ^1^H NMR (600 MHz, D_2_O) δ 5.78 (s, 1H), 5.51 (s, 1H), 3.59 (t, J = 5.9 Hz, 2H), 3.20 (s, 2H), and 1.94 (s, 3H) (see Figure S1a).

### 3.3. Synthesis of 2-Gluconamidoethyl Methacrylamide (GAEMA, Scheme 2 (2))

A GAEMA monomer was prepared through the method reported by Narain et al. with a slight modification [20,33]. D-glucanolactone is an inexpensive and abundant raw material obtained from the oxidation of D-glucose. In this work, to make a solution of D-glucose lactone, it was refluxed at 60 °C for 30 min in methanol, which effectively reduced the reaction time and greatly decreased the amount of chromatographic pure methanol used, making the reaction highly efficient and more environmentally friendly. The specific reaction process was as follows: D-gluconolacton (4 g, 22.45 mmol) was first dissolved in 120 mL methanol and refluxed at 60 °C for 30 min until completely dissolved. A total of 30 mL AEMA (5 g, 30.4 mmol) methanolic solution was subsequently added. The mixed solution changed from clear to milky white and then triethylamine (5.1 mL, 36.6 mmol) was added dropwise. The solution turned from milky white to light yellow and became transparent. The mixture was further stirred at 40 °C for 6 h and permitted to rest. The crude product was subsequently filtered out and washed 3 times with 2-propanol followed by acetone. The product was placed in a vacuum oven at 60 °C for 6 h.

The final yield after purification was 75%. GAEMA was fully characterized by ^1^H and ^13^C NMR. GAEMA: ^1^H NMR [GAEMA: ^1^H NMR (600 MHz, D_2_O) δ 5.62 (s, 1H), 5.38 (s, 1H), 4.22 (d, J = 3.5 Hz, 1H), 4.00 (d, J = 2.7 Hz, 1H), 3.74 (s, 1H), 3.73 (d, J = 2.1 Hz, 1H), 3.67 (d, J = 5.3 Hz, 2H), 3.58 (d, J = 5.8 Hz, 1H), 3.58–3.52 (m, 1H), 3.43–3.36 (m, 1H), 3.36 (dd, J = 11.1, 6.5 Hz, 3H), 3.36–3.32 (m, 2H), 3.32 (d, J = 4.8 Hz, 1H), 1.84 (s, 3H) (see Figure S2a)].

### 3.4. Synthesis of APTES-Modified Porous Silica with Surface Amino Group (SiO_2_-NH_2_, Scheme 2 (3))

Firstly, 5.0 g of 5 µm silica particles were hydrolyzed in 68 mL of 10% hydrochloric acid at 130 °C under stirring reflux for 12 h. The hydrolyzed silica particles were then filtered, washed with water until neutralized, and dried under vacuum at 110 °C for 24 h [34]. Second, 3.0 g of hydrolyzed silica particles, 60 mL of toluene and 6 mL of APTES were placed in a 250 mL flask. After evacuating and filling with Ar three times, the flask was immersed in an oil bath at 115 °C for 24 h. After the end of the reflow, the resulting SiO_2_-NH_2_ was transferred to a sand core funnel and then purified using toluene and methanol to remove excess KH-550. It was then dried in a vacuum oven at 60 °C until a constant weight was obtained.

### 3.5. Preparation of ATRP Initiator-Immobilized Silica Particles (SiO_2_-Br, Scheme 2 (4))

SiO_2_-NH_2_ (3.00 g) dried overnight under vacuum was placed in a dried flask and dispersed in the dry toluene (60 mL); it reacted at room temperature for 1 h. Next, the mixture was cooled to 0 °C. To this, triethylamine (4.5 mL, 32.1 mmol) was added dropwise over 10 min. A solution of 1.5 mL of 12.45 mmol 2-bromoisobutyryl bromide and 5 mL chloroform was added dropwise over 40 min at 0° C. After the addition, the reaction was carried out while stirring at 0° C for another 2 h. The mixture was transferred to the room temperature stirring 24 h. The resultant alkyl bromide functionalized silica particles (SiO_2_-Br) were washed with ethanol five times and the excess ETOH removed by centrifugation (10000 rpm, 10min). The SiO_2_-Br silica was thereafter dried under vacuum at 50° C until the quality remained the same.

### 3.6. Synthesis of SiO_2_-g-poly (2-gluconamidoethyl methacrylamide) Hybrid Microspheres via Surface-Grafted ATRP (Scheme 2 (5))

GAEMA (1.52 g, 5.00 mmol), as the functional monomer, was heated to 60 °C to aid its dissolution in 40.0 mL of methanol/water (3/1, *v*/*v*). The SiO_2_-Br initiator was then added, and this mixture was purged with Ar for 20 min. CuBr (10.0 mg, 0.085 mmol) and PMDETA (35.42 µL, 0.17 mmol) were added, and the resulting dark green solution was stirred under an Ar gas atmosphere. The polymerization mixture was deoxygenated through three freeze–pump–thaw cycles and then immersed into an oil bath preheated to 45 °C and rotated for 24 h. Finally, silica-grafted poly (2-gluconamidoethyl methacrylamide) (SiO_2_-g-GAEMA) was collected by centrifugation (8000 rpm, 5 min) and washed with 2-propanol, methanol, and water, successively, to remove the monomer that was not grafted. The product was dried at 60 °C in a vacuum for 12 h. The greenish-blue filtrate was washed with methanol and then freeze-dried overnight. Unreacted monomers can be used in the next synthesis to maximize monomer utilization.

### 3.7. Calculation of Grafting Ratio of Sugar-Containing Polymer on Silica Gel

The polymer grafting density was calculated from elemental analysis data as interpreted in Table 1. For the prepared graft polymer, the SiO_2_-g-GAEMA stationary phase was calculated using elemental analysis to obtain the graft ratio of SiO_2_-g-GAEMA. The graft ratio of SiO_2_-g-GAEMA was calculated according to the following formula [35] (1):

(1) Grafted pGAEMA=1000CCP, cald.×[1−CPCP, cald.−CiCi, cald.]×A

where C (%) is the percentage of carbon in the polymer; C_P_ (%) is the percentage of carbon added after graft polymerization; C_P, cald._ (%) is the theoretical percentage of carbon in the monomer molecule Content; C_i_ (%) is the percentage of carbon added after fixing the initiator; C_i, cald._ (%) is the theoretical percentage of the upper carbon in the initiator unit molecule; and A is the specific surface area of the silica gel (310 m^2^/g).

### 3.8. Column Packing and Chromatographic Conditions

SiO_2_-g-GAEMA was dispersed in dioxane and packed into a column (150 mm 4.6 mm i.d.) in a balanced density slurry packing technique using methanol as the packing solvent. The newly synthesized column was equilibrated with 90/10 acetonitrile/water for half an hour before each use, and was injected after the baseline was stable. The column was rinsed with 85/15 methanol/water for 2 h and the column was sealed. The efficiency and retention times were determined by HPLC using uracil (200 ppm) as a test substance in reversed phase chromatography mode. The column efficiency of this stationary phase was determined with a uracil probe, acetonitrile (ACN)/water (90/10, *v*/*v*), at a flow rate of 1.0 mL/min, a detection wavelength of 254 nm, and a column temperature of 30 °C.

## 4. Conclusions

A well-structured sugar-containing polymer hybrid filler was prepared through surface initiated ATRP. The initiator was immobilized on the surface of the silica particles and then used as a macroinitiator onto initiate controlled polymerization of atomic radicals on the surface. The amount of GAEMA grafted onto the surface of the silica gel substantially increased with reaction time. Next, the stability of the SiO_2_-g-GAEMA stationary phase was evaluated using four polar compounds (uracil, adenosine, cytosine, and cytidine), and we found that the fixed phase of the grafted sugar-containing polymer had a better peak shape and separation efficiency than the unmodified silica filler. In future research, we will separate sugar-containing systems that combine monosaccharides, disaccharides, and polysaccharides. At the same time, the effect of column temperature and pH of the buffer on the separation efficiency will be considered. For example, the separation of highly isomerized sugar mixtures including pentoses and hexoses is important in the sugar industry.

## Figures and Tables

**Table 1:** Elemental analysis of the Si-OH, Si-NH_2_, Si-Br, and Si-g-GAEMA.

Sample	Content of element		
	**N**	**C**	**H**
Si-OH	0.030	1.003	0.300
Si-NH_2_	2.750	4.906	0.994
Si-Br	1.775	5.070	0.910
Si-g-GAEMA (reaction time = 6 h)	0.937	5.250	0.302
Si-g-GAEMA (reaction time = 12 h)	0.890	7.560	0.357
Si-g-GAEMA (reaction time = 24 h)	2.413	11.523	0.553

**Table 2:** Characterization of GAEMA grafted silica particles with reaction times from 6 to 24 h.

Sample	Reaction time (h)	Grafted (mg/m^2^)
1	6	0.50
2	12	0.78
3	24	1.36

**Table 3:** Column efficiency of SiO_2_-g-GAEMA

Column	Retention time (min)	N (plates/m)	Symmetry factor
SiO_2_-OH	2.687	3960	0.75
SiO_2_-g-GAEMA	2.380	8258	0.98

## Supplementary Materials

Supplementary materials can be found at http://www.mdpi.com/1422-0067/20/1/10/s1. Figure S1: (a) ^1^H NMR spectrum (D_2_O) of AEMA; (b) ^13^C NMR Spectrum (D_2_O) of AEMA; Figure S2: (a) ^1^H NMR spectrum (D_2_O) of GAEMA; (b) ^13^C NMR Spectrum (D_2_O) of GAEMA; Figure S3: shows the C-H in CH_2_ and CH_3_ of FT-IR spectra of (a) bare SiO_2_, (b) SiO_2_-NH_2_, (c) SiO_2_-Br, (d) SiO_2_-g-GAEMA time = 6 h), (e) SiO_2_-g-GAEMA (time = 12 h), and (f) SiO_2_-g-GAEMA (time = 24 h).

## Abbreviations

ATRP	Atom transfer radical polymerization
AEMA	2-Aminoethyl methacrylamide hydrochloride
GAEMA	2-Gluconamidoethyl Methacrylamide
SiO_2_-g-GAEMA	SiO_2_-g-poly (2-gluconamidoethyl methacrylamide)
PMDETA	1,1,4,7,7-pentamethyldiethylenetriamine
