# Supplementary material for: Glycopolymer Grafted Silica Gel as Chromatographic Packing Materials
Source: Int J Mol Sci. 2018 Dec 20;20(1):10. doi: 10.3390/ijms20010010 (PMC6337448; doi:10.3390/ijms20010010)
Supplement: Supplementary file 1 [file ijms-20-00010-s001.pdf]

Article

Glycopolymer Grafted Silica Gel as Chromatographic Packing Materials

Gaoqi, Ma [1,2], Xitao Luo [2,3], Xitong, Sun [2], Weiyan Wang [1,*], Qinghui Shou [2,*], Xiangfeng Liang [2,*] and Huizhou Liu [2]

1 School of Chemical Engineering, Xiangtan University, Xiangtan, Hunan 411105, China; Magq@smail.xtu.edu.cn (G.M.)

2 CAS Key Laboratory of Bio-Based Materials, Qingdao Institute of Bioenergy and Bioprocess Technology (QIBEBT), Chinese Academy of Sciences (CAS), Qingdao 266101, China; luoxt@qibebt.ac.cn (X.L.), sunxt@dicp.ac.cn (X.S.); hzliu@ipe.ac.cn (H.L.)

3 University of Chinese Academy of Sciences, Shijingshan District, Beijing 100049, China

* Correspondence: wangweiyan@xtu.edu.cn (W.W.); shouqh@qibebt.ac.cn (Q.S.); liangxf@qibebt.ac.cn (X.L.); Tel.: +86-532-5856-8201 (Q.S.)

Received: 31 October 2018; Accepted: 18 December 2018; Published: date

AEMA: ^{1}H NMR (600 MHz, D_2O) δ 5.78 (s, 1H), 5.51 (s, 1H), 3.59 (t, J = 5.9 Hz, 2H), 3.20 (s, 2H), and 1.94 (s, 3H). (Figure S1 (a))

GAEMA: ^{1}H NMR [GAEMA: ^{1}H NMR (600 MHz, D_2O) δ 5.62 (s, 1H), 5.38 (s, 1H), 4.22 (d, J = 3.5 Hz, 1H), 4.00 (d, J = 2.7 Hz, 1H), 3.74 (s, 1H), 3.73 (d, J = 2.1 Hz, 1H), 3.67 (d, J = 5.3 Hz, 2H), 3.58 (d, J = 5.8 Hz, 1H), 3.58–3.52 (m, 1H), 3.43–3.36 (m, 1H), 3.36 (dd, J = 11.1, 6.5 Hz, 3H), 3.36–3.32 (m, 2H), 3.32 (d, J = 4.8 Hz, 1H), 1.84 (s, 3H) (see Figure S2 (a))].

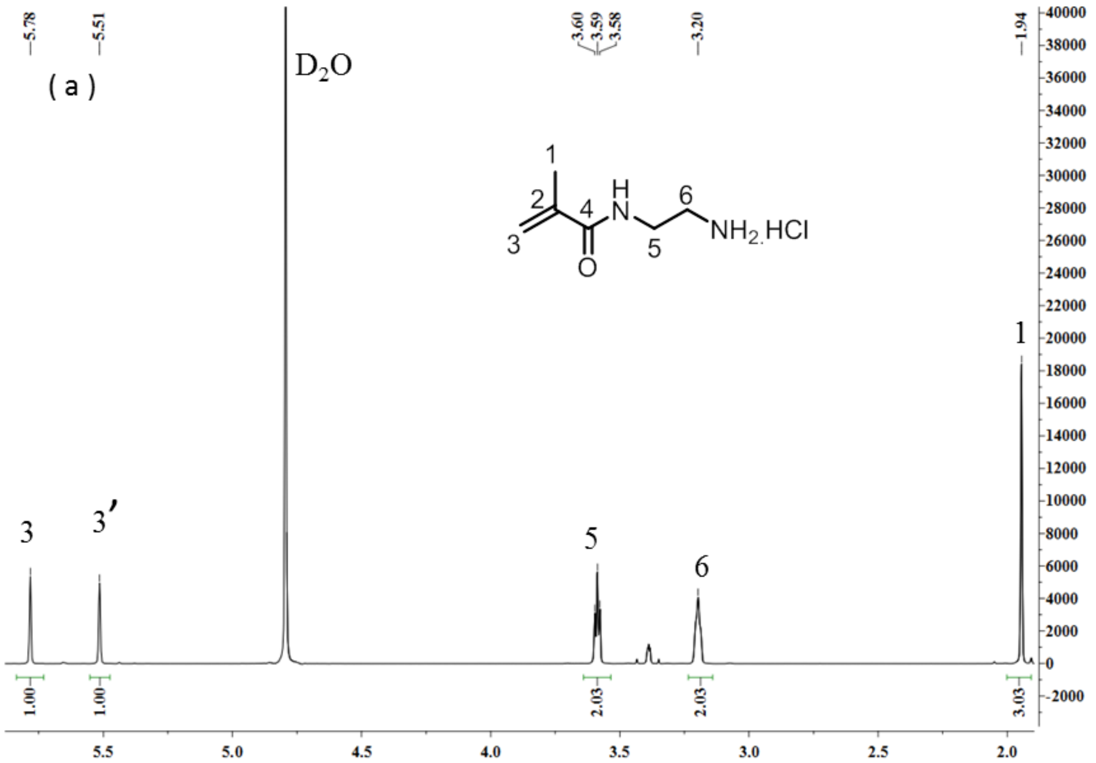

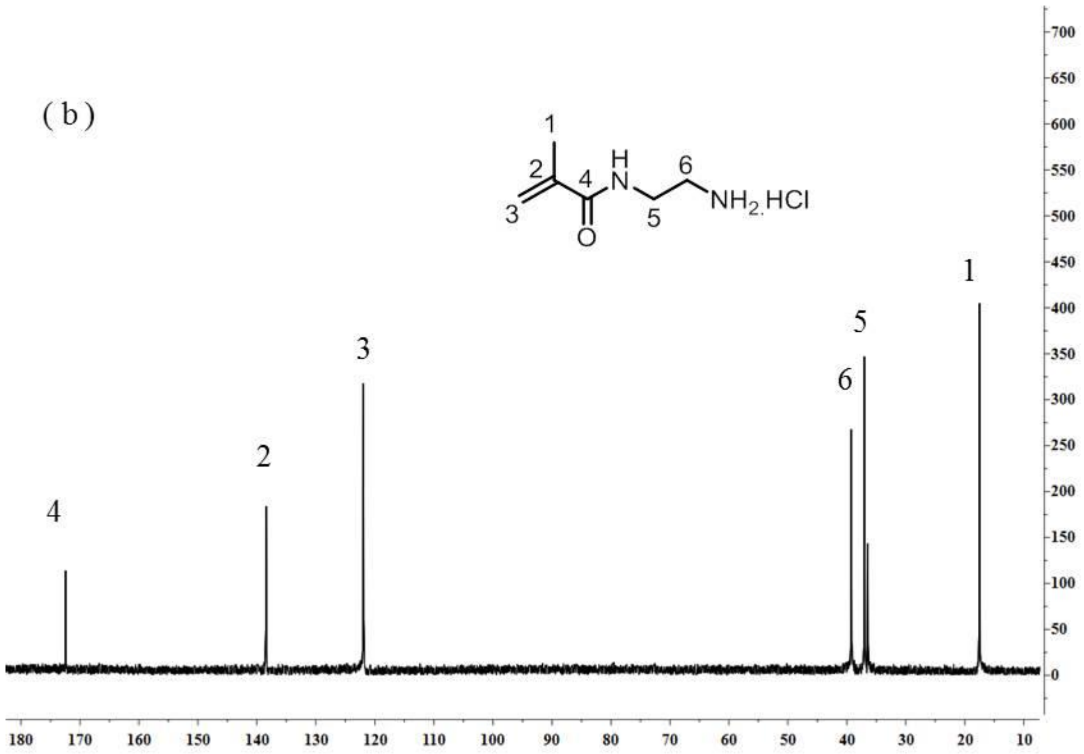

Figure S1. (a) ^{1}H NMR spectrum (D_2O) of AEMA; (b) ^{13}C NMR Spectrum (D_2O) of AEMA.

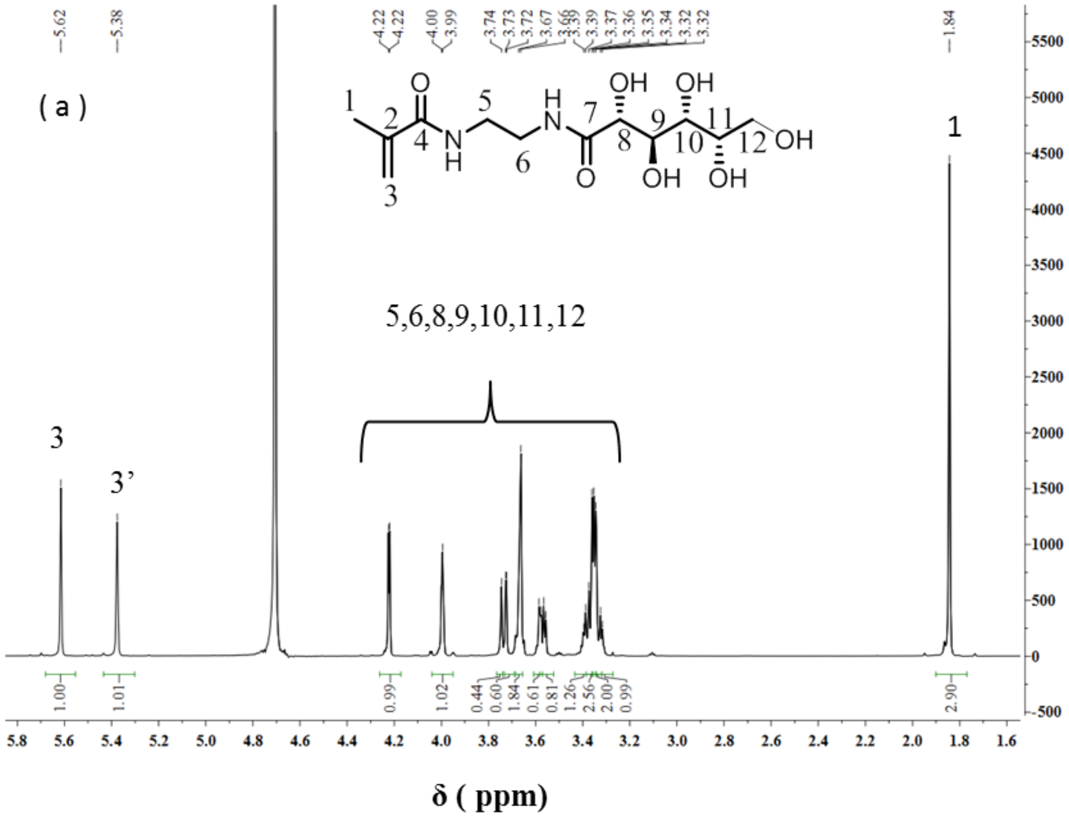

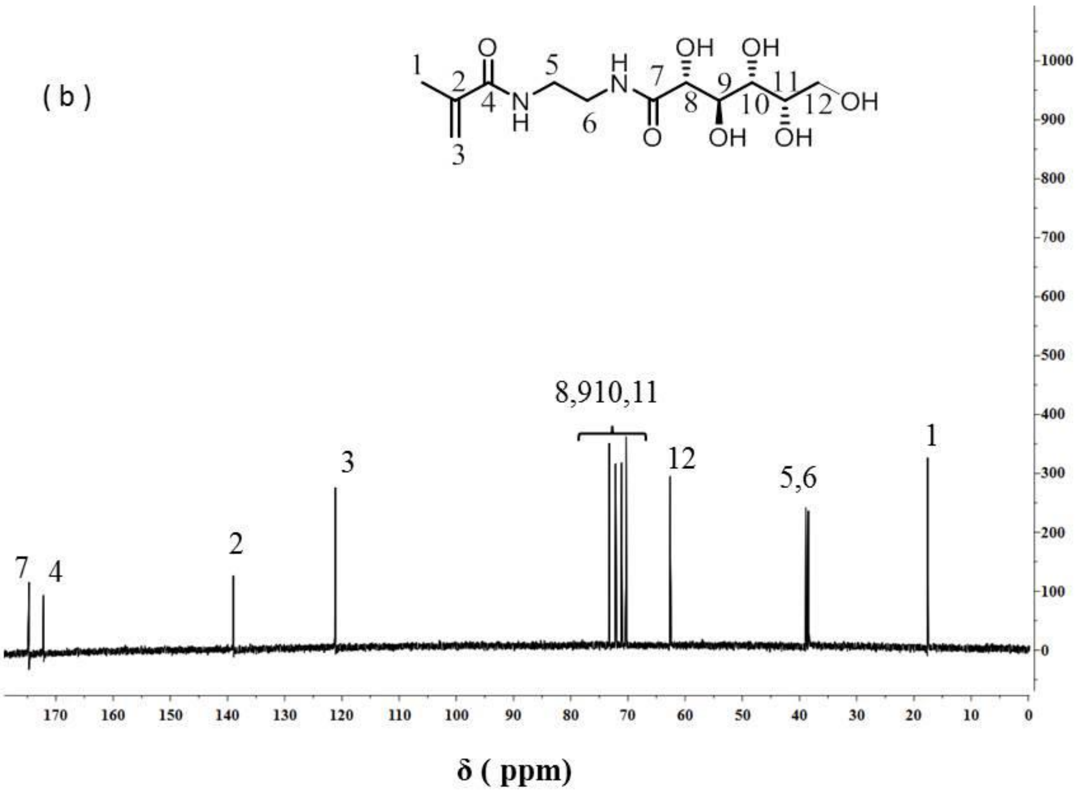

Figure S2. (a) ^{1}H NMR spectrum (D_2O) of GAEMA; (b) ^{13}C NMR Spectrum (D_2O) of GAEMA.

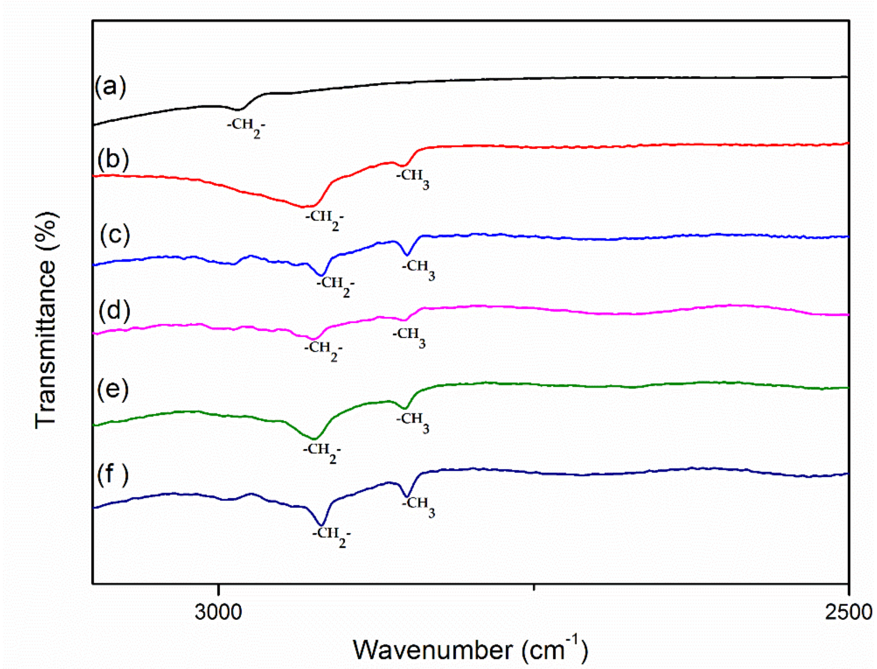

Figure S3. shows the C-H in CH_2 and CH_3 of FT-IR spectra of (a) bare SiO_2, (b) SiO_2-NH_2, (c) SiO_2-Br, (d) SiO_2-g-GAEMA time = 6 h), (e) SiO_2-g-GAEMA (time = 12 h), and (f) SiO_2-g-GAEMA (time = 24 h).
